# The Practical Medicine Series of Year Books

**Published:** 1903-05

**Authors:** 


The Practical Medicine Series of Year Books. Ten volumes. Issued monthly, under the general editorial charge of Gustavus P. Head, M. D., Professor of Laryngology and Rhinology in the Chicago Post-Graduate Medical School. Volume X. Skin and Venereal Diseases, Nervous and Mental Diseases. Edited by W. L. Baum, M. D., and Hugh S. Patrick, M.D. Duodecimo, pp. 245. Chicago: The Year Book Publishers. 1902. [Price, $1.25; entire series, $7.50.]

Volume X. of the Practical Medicine Series is devoted to skin and venereal diseases and to nervous and mental diseases.

These year books have become very popular with the profession and deserve all the success attained. The several departments are edited by competent writers and the literature of the world is succinctly reviewed. The part devoted to nervous and mental diseases covers an unusually wide field of literature, and the review and annotations made by Dr. Patrick are most useful and instructive. The index is carefully compiled and enables the reader to seek quickly the desired information. The price of the volume has been reduced to $1.25. W.C.K.

